# Optimization of the viability of stem cells derived from umbilical cord blood after maternal supplementation with DHA during the second or third trimester of pregnancy: study protocol for a randomized controlled trial

**DOI:** 10.1186/1745-6215-15-164

**Published:** 2014-05-10

**Authors:** Irene Martini, Enea Gino Di Domenico, Roberta Scala, Francesca Caruso, Carla Ferreri, Filippo M Ubaldi, Andrea Lenzi, Herbert Valensise

**Affiliations:** 1 SmartBank, the stem cells bank, Rome, Italy; 2 Istituto Pasteur-Fondazione Cenci Bolognetti, Department of Biology and Biotechnology “Charles Darwin”, Sapienza University of Rome, Rome, Italy; 3 Department of Obstetrics and Gynaecology, Fatebenefratelli Isola Tiberina Hospital, Rome, Italy; 4 ISOF, National Research Council, Bologna, Italy; 5 GENERA, Centre for Reproductive Medicine, Clinica Valle Giulia, Rome, Italy; 6 Department of Experimental Medicine, Sapienza University of Rome, Rome, Italy

## Abstract

**Background:**

Umbilical cord blood (UCB) is an important source of hematopoietic stem cells (HSCs). However, the concentration of cells in cord blood units is limited and this may represent the main restriction to their therapeutic clinical use. The percentage of metabolically active stem cells provides a measure of the viability of cells in an UCB sample. It follows that an active cellular metabolism causes a proliferation in stem cells, offering an opportunity to increase the cellular concentration. A high cell dose is essential when transplanting cord stem cells, guaranteeing, in the receiving patient, a successful outcome.

This study is designed to evaluate the impact of docosahexaenoic acid (DHA) supplementation in pregnant women, in order to increase the quantity and viability of the cells in UCB samples.

**Methods/design:**

The metabolic demand of DHA increases in the course of pregnancy and reaches maximum absorption during the third trimester of pregnancy. According to these observations, this trial will be divided into two different experimental groups: in the first group, participants will be enrolled from the 20th week of estimated stage of gestation, before the maximum absorption of DHA; while in the second group, enrolment will start from the 28th week of estimated stage of gestation, when the DHA request is higher. Participants in the trial will be divided and randomly assigned to the placebo group or to the experimental group. Each participant will receive a complete set of capsules of either placebo (250 mg of olive oil) or DHA (250 mg), to take one a day from the 20th or from the 28th week, up to the 40th week of estimated gestational age. Samples of venous blood will be taken from all participants before taking placebo or DHA, at the 20th or at the 28th week, and at the 37th to 38th week of pregnancy to monitor the level of DHA. Cell number and cellular viability will be evaluated by flow cytometry within 48 hours of the UCB sample collection.

**Trial registration:**

International Standard Randomised Controlled Trial Number Register: ISRCTN58396079. Registration date: 8 October 2013.

## Background

Umbilical cord blood (UCB) cells provide an alternative source of hematopoietic stem cells (HSCs) for those patients with hematologic diseases unable to find a fully compatible donor.

Over the last 25 years, the field of UCB banking and transplantation has grown exponentially [1]. However, despite the successes, one of the main problems with using UCB for transplantation is related to the reduced number of hematopoietic progenitor cells (HPCs) and HSCs compared with bone marrow or mobilized peripheral blood grafts.

The risk of graft failure increases with the limited number of available HPCs and HSCs, including delayed hematopoietic engraftment [2-4] and delayed immune reconstitution [5,6]. The studies show a cumulative incidence of nonengraftment that ranges from 10 to 20% after UCB transplantation, while the median time for neutrophil recovery is between 22 and 27 days.

Protocols on intravenous CD34+ stem cell transplants specify a minimum dose equivalent to 3 × 10^7^ mononuclear cells/kg of body weight and 1.5 × 10^5^ CD34+/kg of the patient’s weight at the time of the infusion. Umbilical cord collections are 11 × 10^8^ mononuclear total number of cells and 3 × 10^6^ CD34+, thus usually sufficient for a transplant in a 14-year-old child [7].

It is evident that it is necessary to increase the proliferative ability of stem cells in the UCB so that these cells are viable and sufficiently mature to engraft after transplant.

**Figure 1:**
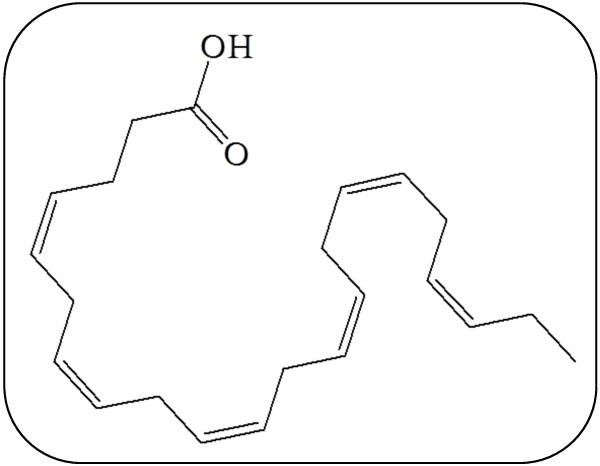
Chemical structure of docosahexaenoic acid (DHA).

*In vitro* experiments showed that the viability of the HSCs, deriving from UCB, could be augmented with favourable effects on the engraftment potential [8,9]. These studies revealed that the beneficial effect of the *in vitro* generation of megakaryocytes from UCB, together with a higher ability to survive the cryopreservation, were observed when docosahexaenoic acid (DHA) and arachidonic acid (AA) were supplemented in the growth medium [8,9], increasing the clinical utility of the UCB [9]. In particular, DHA, which belongs to the polyunsaturated fatty acids (PUFAs) family, is a fundamental element of cell membranes [10,11], especially those of the brain and retina [12]. The chemical structure of DHA is illustrated in Figure 1. DHA accumulation in the brain and retina occurs mainly between the last intrauterine trimester and the initial months of life. The metabolic demand of DHA increases progressively in the course of pregnancy, particularly during the third trimester. In this phase a sudden acceleration of foetal growth is observed together with a great deposition of fatty acids in the central nervous system [13]. Therefore, the level of DHA during pregnancy can directly influence the stages of development [14]. Observational studies have also demonstrated a close association between the DHA availability during the perinatal period with long-term cognitive and visual development [15,16]. Besides, the consumption of large quantities of fish, rich in DHA, has been associated with an increase in gestational age, a greater foetal weight at birth and less incidence of pre-eclampsia [17,18].

The effect of DHA on foetal weight and development is linked to its effect on the endothelium. DHA increases the flow of blood through vasodilatation in young adults and improves membrane receptor activity. Therefore, it is possible that these fatty acids improve foetal growth, increasing the placental flow by potentiating the activity of prostacyclin versus thromboxane and reducing blood viscosity [19].

Furthermore, treatment with DHA in the third trimester of the pregnancy improves the mother’s DHA status, as indicated by the composition of fatty acids in the maternal erythrocyte membrane [20-22].

However, it should be considered that, during the course of the third trimester, the foetus absorption of DHA is 70 to 100 mg from the maternal bloodstream daily; at the same time, during the second half of the pregnancy, the mother’s needs of DHA supplementation also grows to support the higher request from the foetus without any detrimental effect on her own reserves. Hence, during the third trimester, with an excess or greater availability of DHA, the foetus will tend to accumulate it preferentially in the brain tissue competing with the accumulation on the surface of other cells, specifically, HSCs.

In Denburg *et al*. [23], the effects of a supplementation with n-3 PUFA were assessed in a population of neonatal CD34+ cells as a method for preventing allergies. In this study, the pregnant women were treated, from the 20th week of gestation until delivery, with fish oil capsules or a placebo of olive oil capsules in a randomized control trial (RCT). The results revealed that, in the group given fish oil, the percentage of CD34+ present in the cord blood was greater than that found in the control group (0.879 ± 0.096 versus 0.533 ± 0.041; *P* <0.002) [23]. The study confirmed that a maternal dietary supplementation with n-3-PUFA increases the levels of these fatty acids in the infant’s cell membranes and this has a significant effect on the CD34+ cell population in the cord blood.

The aim of this study is to increase the viability of the stem cells collected at birth from the UCB, in particular of the haematopoietic lineage, by administrating purified DHA during the second (20th week of estimated gestational stage) or third (28th week of estimated gestational stage) trimester of pregnancy. The purified DHA consists of at least 250 mg/day derived from purified fish oil or from microalgae extraction administered to pregnant women orally in the form of capsules in a randomized, double-blind, monocentric study. The choice to administer 250 mg/day is prompted by the European Community’s recommended dose for treatment in pregnancy, based on the outcomes of the Perinatal Lipid Intake Working Group and the Early Nutrition Programming Project. This dose is sufficient to maintain maternal-foetal unity and satisfy the needs of both without incurring the risk of a deficiency in this nutrient.

In this trial, we hypothesize that a DHA supplementation, during the second or third trimester of pregnancy, can increase the metabolic activity of the stem cells in UCB samples overcoming the limit imposed by the extraction of cord blood. Indeed, unlike other sources, UCB cells have only one possibility of extraction, and it is thus essential that the cell product obtained is qualitatively and quantitatively sufficient for a transplant in adulthood. This treatment can also foster the engraftment of stem cells of the haematopoietic lineage after transplant, reducing the window period after transplantation in which haematopoietic stem cells must colonize the bone marrow, and specialize into cells of the circulatory system and of the immune system.

## Methods

### Recruitment

The trial is designed to target pregnant women within the 20th or 28th week of gestation. At the time of recruitment, the participants will sign an informed consent form, and complete a questionnaire authorizing third parties to obtain demographic, anamnestic and nutritional data. The information thus obtained will be entered into a case report form for each pregnant woman.

Detailed information on benefits and risks of DHA will be provided to study candidates. Participants that meet pre-specified eligibility criteria (Table 1) and provide written informed consent will be enrolled in the study.

**Table 1:** Eligibility criteria

**Inclusion criteria**	**Exclusion criteria**
Caucasian	When it is impossible to collect cord blood (bureaucratic reasons or because of emergencies regarding the health of the mother or the baby)
Non-smokers	Women who have taken other supplements containing DHA or fish oil
Over 18 years of age	Mother with a temperature of 39°C during delivery
Single pregnancy	UCB samples with a volume of less than 80 ml and/or less than 70% cell vitality
Absence of diabetes or hypertension, or any other type of pathology requiring pharmacological therapy	
Absence of chromosome abnormalities and/or congenital malformations in the foetus	
HBV, HCV, HIV and CMV negative	

CMV, cytomegalovirus; DHA, docosahexaenoic acid; HBV, hepatitis B virus; HCV, hepatitis C virus; UCB, umbilical cord blood.

The Ethics Committee of the San Giovanni Calibita Fatebenefratelli Hospital (Rome, Italy), in compliance with the Helsinki Declaration, approved the study protocol (9 July 2009, reference: 35/2009; and 9 December 2010, reference: 67/2010). The trial is registered at the International Standard Randomised Controlled Trial Number Register (ISRCTN58396079).

### Study design

**Figure 2:**
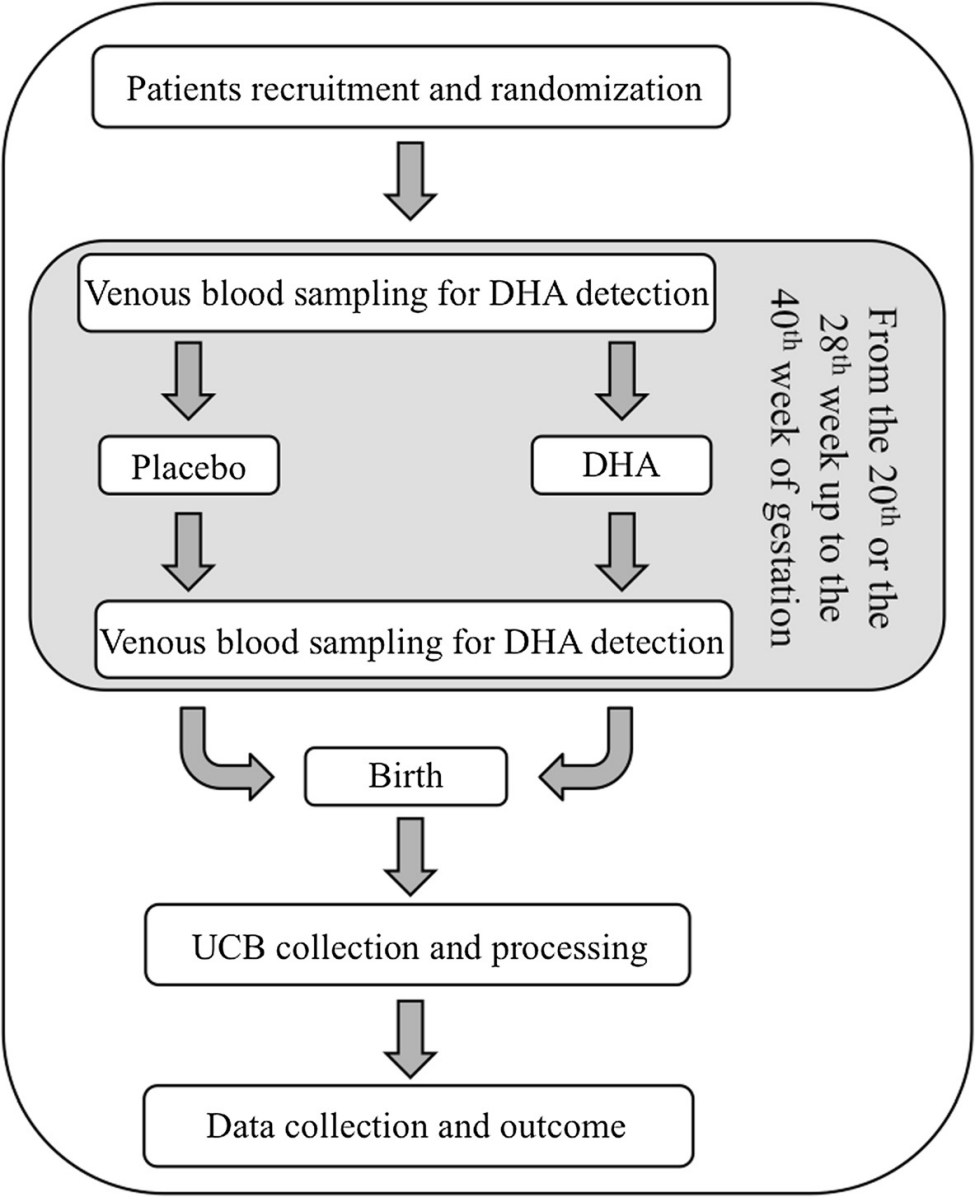
**Work flow for the study.** After recruitment the participants will be randomized into one of the two groups. Samples of venous blood will be taken from all participants before taking placebo or DHA (20th or 28th week) and at the 37th to 38th week of pregnancy to monitor the level of DHA. After birth, UCB samples will be collected and processed within 48 hours. DHA, docosahexaenoic acid; UCB, umbilical cord blood.

Participants included in the trial will be divided into two groups: one group will be given the placebo; and the other group will be given DHA (Figure 2). The placebo or DHA will be assigned in a double-blind, randomized manner, created by a computer program producing a sequence of codes attributed to one of the two groups. An identifying code, assigning the woman to one of the two study groups, guarantees, from that moment on, anonymity in treating sensitive data.

Each participant will receive a complete set of capsules of either placebo or DHA, to take one a day from the 20th or 28th week up to the 40th week of estimated gestational age. The placebo pills (placebo batch) will contain 250 mg of olive oil. This choice is conditioned by the need to use a product of proven safety with a consistency and flavour as similar as possible to fish oil.

The DHA capsules (active batch) will contain 250 mg of DHA (Incromega™ DHA 250TG SR, Croda, Goole, UK) + 10 mg natural vitamin E (Avantgarde sigma-tau, Rome, Italy). The DHA in the capsule is extracted from fish oil by PureMax™ technology (Croda) that enables lipids to be purified and concentrated to maximum standards. This ensures that the DHA used for this study is free of contaminants, including: heavy metals, such as mercury, arsenic, cadmium and lead; oxidant impurities; dioxins and furans; dioxin-type polychlorinated biphenyls; and polycyclic aromatic hydrocarbons.

The packaging of the capsules in the two groups will be identical (see Table 2).

**Table 2:** Features of the DHA and placebo batch

**Feature**	**DHA**	**Placebo**
Tablet formulation	250 mg Incromega DHA + 10 mg natural vitamin E	250 mg olive oil
Capsule type	10 mm, oval shape	10 mm, oval shape
Capsule colour	Dark yellow	Dark yellow
Batch production	20,000 tablets	20,000 tablets
Size of the blisters	82 × 132 mm	82 × 132 mm
Blister per case	1	1
Number of tablets per blister	30	30
Packaging	White box	White box

DHA, docosahexaenoic acid.

### Evaluation of erythrocyte membrane DHA levels

To evaluate the effectiveness of the diet supplement, a venous blood sample will be taken from half of the study participants with the purpose of finding the DHA concentration in the erythrocyte membranes. Two samples of 2 cc of venous blood will be taken from all participants before taking placebo or DHA, at the 20th or 28th week after recruitment, and at the 37th to 38th week of pregnancy. The blood samples will be taken with a specific kit provided by Lipinutragen-CNR (Bologna, Italy) and shipped to the Lipinutragen-CNR laboratory for testing. The DHA concentration, with respect to the total erythrocyte membrane phospholipids, will be determined by treating a 0.5 ml portion of the maternal blood sample or cord blood samples to separate the erythrocytes and isolate the erythrocyte membranes [24]. The fraction of total phospholipids will be treated with 0.5 M KOH/MeOH for 10 minutes at room temperature in order to obtain the chemical derivation of the phospholipids and compare them with the corresponding chemical fatty acid methyl esters (FAME). FAMEs will be evaluated by gas chromatography (GC) with a flame ionization detector (Agilent 6850; Agilent Technologies, Santa Clara, CA, USA) and a DB-23 column (90% biscyanopropyl/10% phenylcyanopropyl polysiloxane capillary column; 60 m, 0.25 mm ID, 0.25 μm film thickness) or similar, with hydrogen as carrier gas at a constant flow of 1 ml/min; the oven programme will be fixed for the efficient separation of the FAMEs, including the cis and trans isomers. FAMEs can be recognized by comparing them with commercially available references. Trans geometric isomers will be recognized by comparison with a library of trans isomers obtained by radical isomerization as described in previous publications [25]. The FAMEs will be identified by a randomized analysis of the samples by GC coupled with mass spectrometry. The values of the fatty acids will be given as a percentage of all the most important peaks resulting from the GC analysis, representing 98 to 99% of the total chromatographic peaks. The analyses will be repeated so that the value of the DHA can be checked at zero time and compared with the known normal range in healthy subjects.

The statistical assessment of the treatment effectiveness will be obtained by comparison with the DHA values obtained after therapy.

### Separation of CD34+ protocol

UCB cells will be stained with fluorescein isothiocyanate (FITC)-conjugated monoclonal antibody against CD45+ and phycoerythrin (PE)-conjugated monoclonal antibody against CD34+. These will be used to identify leukocytes and HSCs, respectively. For analysis of the viability of the cells, they will also be stained with 7-aminoactinomycin D (7-AAD), which will bind to the DNA of cells that are undergoing or have undergone apoptosis (Stem Kit™ Beckman Coulter, Brea, CA, USA). The stained cells will then be lysed (1:10 dilution of ammonium chloride 10x concentrated and deionized water) and analyzed by flow cytometer with a FC 500 cytometer (Beckman Coulter) using CXP software under a single ISHAGE gating strategy.

### Collection of cord blood

Before extracting the cord blood, the recruited pregnant women must carry out serological tests to prove the absence of the following infectious diseases: hepatitis B virus; hepatitis C virus; HIV; cytomegalovirus; human T-cell lymphotropic virus; and syphilis. The report will then be presented to the hospital to obtain the licence.

All the participants will receive a free cord blood collection kit provided by SmartBank (SmartBank, Rome, Italy). Hospital staff assisting the birth will immediately extract and collect the cord blood with the kit. Samples will be shipped to the Biovault technical laboratory (Plymouth, UK) for specific analysis of: volume (ml); pH; cell viability (%); number of CD34+ cells; number of leukocytes; and bacterial and viral tests. In order to determine the DHA percentage in the cord blood erythrocyte membranes, 2 cc of the extracted cord blood will be sent to the Lipinutragen-CNR laboratory in Bologna.

### Processing of cord blood

The cord blood processing procedures will be carried out in the Biovault laboratory in the UK, possessing medical and healthcare products, and regulatory agency registration, operating according to the World Health Organization (WHO) international good manufacturing practices protocol, and the International Organization for Standardization (ISO) ISO 9001:2008 and ISO 13485:2012 standards, and aseptic treatment, grade A certificate, will be performed in a Biosafety Level 3 (BSL-3) clean room. Biovault is accredited by the American National Standards Institute (ANSI) regulatory accrediting body, Advancing Transfusion and Cellular Therapies Worldwide, and the Joint Accreditation Committee of the International Society for Cellular Therapy (ISCT) and the European Group for Blood and Marrow Transplantation (EBMT) (JACIE), and has a Human Tissue Authority licence.

The sample will enter the BSL-3 clean room through a hermetically sealed window with positive outlet atmospheric pressure. Inside the BSL-3 clean room the sample will be weighed, centrifuged, measured volumetrically, and subjected to microbiological tests, flow cytometry analysis and phase separation with subsequent selection of mononuclear cells. The sample containing the cells will be inserted into the final Pall container (Pall Corporation, Port Washington, NY, USA) and mixed at 4°C with 10% dimethyl sulfoxide (DMSO). The bag or Pall, with a unique, indelible identification code, will be inserted into a computerized temperature reduction system. After the temperature is reduced, the Pall will be inserted in a sealed metal container and preserved in liquid nitrogen vapour at -196°C.

### Flow cytometry

The flow cytometry analysis will be carried out within 48 hours of the UCB sample collection with a FC 500 flow cytometer (Beckman Coulter) on ISHAGE software platform. The analysis method uses double fluorescence (FITC-PE) with rat monoclonal antibody. For a simultaneous identification and count of the CD45+ and CD45+ CD34+ cells, a double antibody marking will be carried out, evaluating the percentage of the positive population with regards to the absolute count. The CD34+ receptor will be used as an indicator of the total number of progenitor stem cells of the haematopoietic lineage. The cell viability will be evaluated by flow cytometry using 7-AAD (Invitrogen Life Technologies, Carlsbad, CA, USA). Apoptotic, necrotic and metabolically inactive cells will be stained and thus considered non-viable while viable cells remain unstained. Total cells will be counted by flow cytometry discriminating between non-viable (7-AAD positive cells) and viable cells (7-AAD negative cells). The flow cytometer will be calibrated every 24 hours and quality controls performed according to the UK National External Quality Assessment Service (NEQAS) protocol.

### Statistical analysis

The statistical analysis of the data obtained will be carried out with the aid of the Statistical Package for the Social Sciences (SPSS) program (IBM Corporation, Armonk, NY, USA) in cooperation with the Fatebenefratelli Association for Research (AFaR) of the San Giovanni Calibita Fatebenefratelli Hospital (Rome, Italy). Each experimental group will be compared versus the respective placebo group. The effectiveness of the DHA supplementation at different stages of gestation will also be evaluated by comparing the two experimental groups. The differences between the quantitative variables of the two groups will be analyzed with the Student’s *t*-test and analysis of variance (ANOVA); results of *P* <0.05 will be considered statistically significant. The Spearman test will also be used to compare the parameters of interest, and other quantitative and non-quantitative variables presented by the groups studied.

## Discussion

The proposed study is a randomized, double-blind, placebo-controlled, clinical trial designed to investigate the possible positive effects on the quantity and viability of stem cells in UCB samples, in pregnant women supplemented with a dose of 250 mg/day of DHA, from the 20th or from the 28th week of pregnancy. No prospective data is presently available; however, if the study shows benefits, in terms of the quality and quantity of the stem cells, DHA could be regularly administered to pregnant women to potentiate the chances of success of transplants from UCB.

### Trial status

Patient recruitment is currently being undertaken.

## Abbreviations

7-AAD: 7-Aminoactinomycin D; ANSI: American National Standards Institute; ANOVA: Analysis of variance; AA: Arachidonic acid; BSL-3: Biosafety Level 3; CMV: Cytomegalovirus; DMSO: Dimethyl sulfoxide: DHA: Docosahexaenoic acid; EBMT: European Society for Blood and Marrow Transplantation; FAME: Fatty acid methyl ester; FITC: Fluorescein isothiocyanate; GC: Gas chromatography; HBV: Hepatitis B virus; HCV: Hepatitis C virus; HPC: Hematopoietic progenitor cell: HSC: Hematopoietic stem cell; ISO: International Organization for Standardization; ISCT: International Society for Cellular Therapy; JACIE: Joint Accreditation Committee of the ISCT and EMBT; NEQAS: National External Quality Assessment Service; PE: Phycoerythrin; PUFA: Polyunsaturated fatty acid; RCT: Randomized control trial; SPSS: Statistical Package for the Social Sciences; UCB: Umbilical cord blood; WHO: World Health Organization.

## Competing interests

The authors declare that they have no competing interests.

## Authors’ contributions

IM and HV conceived and designed the study, performed data collection and analysis, and wrote the manuscript. HV, IM and ED monitored the study, logistics, communication with the ethics committee and coordination between study centres. ED, FC and RS performed data collection and contacted people at the study centres. HV, CF and Biovault provided infrastructure provision. FU and AL designed and coordinated the study. All authors read and approved the final manuscript.
